# 980. Risk Factors for Central Line Associated Bloodstream Infection Prior to and During the COVID-19 Pandemic

**DOI:** 10.1093/ofid/ofad500.035

**Published:** 2023-11-27

**Authors:** Jennifer Priem, Mindy Sampson, Shelley Kester, Werner Bischoff, Catherine Passaretti, Casey Stephens

**Affiliations:** Atrium Health, Charlotte, North Carolina; Wake Forest University School of Medicine, Charlotte, North Carolina; Atrium Health, Charlotte, North Carolina; Wake Forest University School of Medicine, Winston Salem, NC; Advocate Health, Charlotte, NC; Atrium Health, Charlotte, North Carolina

## Abstract

**Background:**

Central line associated bloodstream infections (CLABSIs) increased during the COVID-19 pandemic1-3, especially in patients infected with SARS-CoV-2,4 but there has been limited analysis on other risk factors associated with this increase.

**Methods:**

We conducted a retrospective cohort study to assess risk factors for developing a CLABSI prior to (1/2019-3/2020) and during the COVID-19 pandemic (3/2020-2021) across 12 acute care hospitals in the Southeastern U.S. A multivariable logistic regression analysis was performed comparing patients with and without CLABSI.

**Results:**

Of the 46,259 patients with central lines, 313 developed a CLABSI during the study period (109 pre- and 204 during- pandemic, a significant proportional increase.) Risk factors for CLABSI in the pre-pandemic period included cancer diagnosis, receipt of total peripheral nutrition (TPN) during hospital stay, Intensive Care Unit encounter, and longer length of stay and average line days (Table 1). During the pandemic, cancer diagnosis was no longer significantly associated with risk of CLABSI, but additional risk factors included Medicare insurance (OR 1.68), presence of a dialysis catheter (OR 1.57), COVID-19 diagnosis (OR 2.14), higher body mass index (OR 1.01) and Black race (OR 1.36). During the pandemic, we found that Black patients without COVID were 1.6 times (95% CI 1.09, 2.41) more likely to develop CLABSI compared to White patients (Table 2).

**Figure:**
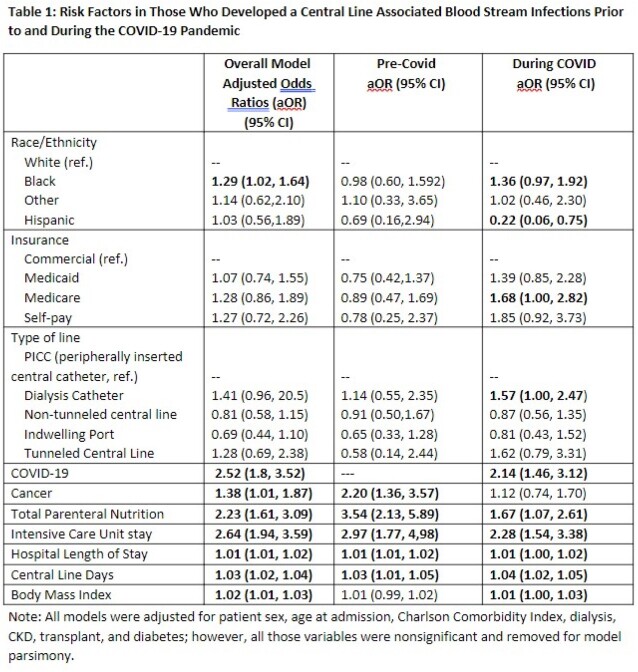

**Figure:**
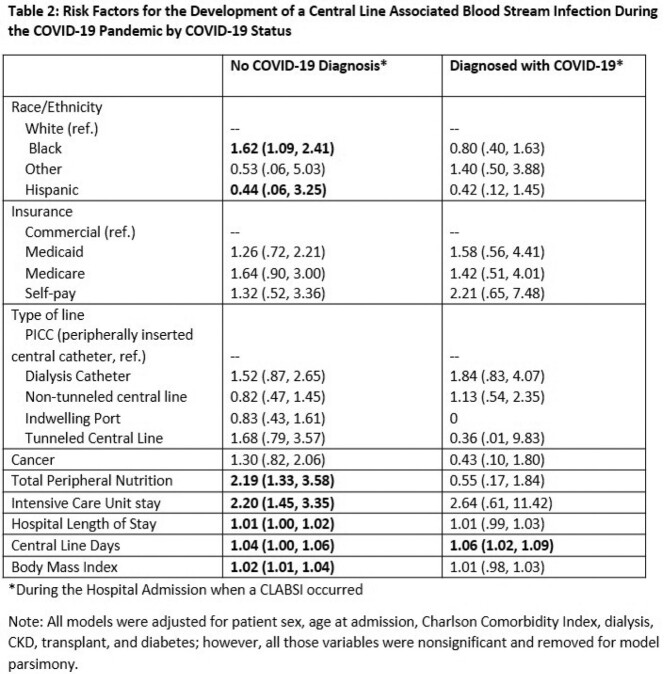

**Conclusion:**

We detected a shift in risk factors for CLABSI during the pandemic. While COVID-19 diagnosis increased the risk of CLABSI, as has been shown in other studies4, our data additionally revealed a shift in risk factors in patients without COVID-19 showing a higher risk in those with black race, Medicare for insurance and presence of a dialysis catheter during the pandemic. These results suggest that alterations may have occurred in healthcare delivery, specifically in vulnerable populations, laying the foundation for future investigations into potential care improvements.

**Disclosures:**

**All Authors**: No reported disclosures

